# Serum copeptin predicts atrial fibrillation recurrence and is associated with increased all-cause mortality

**DOI:** 10.3389/fcvm.2026.1859765

**Published:** 2026-08-25

**Authors:** Martin Ihnatko, Zora Lazúrová, Ivana Jochmanová, Ľudmila Ihnatková, Ivica Lazúrová

**Affiliations:** 1 1st Department of Internal Medicine, Faculty of Medicine, Pavol Jozef Šafárik University in Košice, Košice, Slovakia; 2 4th Department of Internal Medicine, Faculty of Medicine, Pavol Jozef Šafárik University in Košice, Košice, Slovakia; 3 Department of Hematology and Oncohematology, Faculty of Medicine, Pavol Jozef Šafárik University in Košice, Košice, Slovakia

**Keywords:** atrial fibrillation, biomarkers, copeptin, mortality, recurrence

## Abstract

**Introduction:**

Copeptin, a stable surrogate marker of arginine vasopressin, reflects neurohumoral activation and hemodynamic stress. Its prognostic value in heart failure is established, but its role in atrial fibrillation (AF) recurrence and mortality remains unclear. This study aimed to compare copeptin concentrations in patients with AF and controls, evaluate biochemical and echocardiographic associations, and assess copeptin associations with 12-month AF recurrence and all-cause mortality.

**Methods:**

This prospective observational study included 100 hospitalized patients with AF and 50 controls in sinus rhythm. Participants underwent clinical evaluation, echocardiography, biochemical testing, and measurement of copeptin, N-terminal pro-B-type natriuretic peptide (NT-proBNP), and high-sensitivity cardiac troponin T (hsTnT). AF recurrence was evaluated in 62 patients after sinus-rhythm restoration; mortality was assessed in all 100 patients over a 12-month follow-up period.

**Results:**

Median copeptin concentration was higher in patients with AF than in controls, 14.52 (6.98–36.83) versus 4.10 (2.70–6.88) pmol/L (*p* < 0.0001), and remained higher after adjustment for age and sex (adjusted geometric mean ratio, 2.995; 95% CI, 2.040–4.398; *p* < 0.0001). In patients with AF, copeptin correlated positively with C-reactive protein (ρ = 0.4862), hsTnT (ρ = 0.4904), NT-proBNP (ρ = 0.3458), left atrial volume index (LAVi; ρ = 0.2598), and left ventricular mass index (ρ = 0.2252), and inversely with estimated glomerular filtration rate (ρ = −0.3231) and tricuspid annular plane systolic excursion (ρ = −0.2277; all *p* < 0.05). Each 10-pmol/L copeptin increase was independently associated with 61% higher AF recurrence odds after adjustment for age, LAVi, and left ventricular ejection fraction (adjusted OR, 1.61; 95% CI, 1.01–2.56). Copeptin showed modest discrimination for AF recurrence (AUC, 0.674; 95% CI, 0.526–0.821); the 11.68-pmol/L cut-off yielded 63.33% sensitivity and 75.00% specificity. Median copeptin concentration was higher in non-survivors than survivors, 35.35 (10.35–56.82) versus 12.20 (6.54–29.43) pmol/L (*p* = 0.0051).

**Discussion:**

In hospitalized patients with AF, copeptin is elevated and associated with cardiac biomarkers, inflammation, and structural and functional echocardiographic abnormalities. Higher copeptin independently predicts AF recurrence after sinus-rhythm restoration and is associated with 12-month all-cause mortality, supporting its potential for risk stratification and prognostic assessment.

## 1 Introduction

Copeptin is a glycosylated polypeptide that represents the C-terminal part of the pre-prohormone pre-provasopressin and is released through the hypothalamic–pituitar*y* axis together with neurophysin II and arginine vasopressin (AVP). AVP participates in the regulation of water and electrolyte homeostasis and plays an important role in the pathogenesis of cardiorenal dysfunction, while copeptin reflects activation of the vasopressin system (1, 2). Compared with AVP, copeptin is structurally more stable and is released into the bloodstream in equimolar amounts with AVP. Owing to its greater stability, it serves as a surrogate biomarker of AVP secretion (3, 4). It is well known that some extracardiac biomarkers, such as copeptin and adrenomedullin, are involved in cardiovascular humoral regulation (2). Copeptin concentrations increase in patients with heart failure (HF) in response to hemodynamic and neurohumoral stress, including reduced cardiac output and hypotension (5, 6). Its prognostic value in HF has been widely documented (7–10).

However, less is known about the relationship between copeptin and atrial fibrillation (AF), particularly AF recurrence, and the available evidence remains limited and inconsistent (1, 3, 4). AF is the most common sustained cardiac arrhythmia and an important cause of cardioembolic stroke (1). Previously, two natriuretic peptides, brain natriuretic peptide (BNP) and N-terminal prohormone of brain natriuretic peptide (NT-proBNP), have been investigated as biomarkers of AF (11–13). Lang et al. reported that elevated serum copeptin concentrations were associated with a higher risk of a composite cardiovascular endpoint including stroke and death in patients with non-valvular AF (14). Avci et al. also found higher copeptin concentrations in patients with rheumatic mitral stenosis and paroxysmal AF (3). In addition, Ding et al. demonstrated higher serum copeptin concentrations in patients with AF than in healthy controls, with the highest concentrations observed in patients with concomitant acute HF and AF (15).

On the other hand, Arbault-Biton et al. reported that serum copeptin concentrations were not independently associated with AF duration after multivariable adjustment (4). Moreover, the study by Philippsen et al. demonstrated no significant association of circulating cardiac humoral biomarkers, including BNP, troponin I, and copeptin, with incident subclinical AF in patients with diabetes mellitus and hypertension (16). In another study, copeptin, along with other biomarkers, showed no predictive value for AF. In that study, only serum BNP concentration was identified as a significant predictor of AF, with an odds ratio (OR) of 5.96 (*p* = 0.045) (17). However, the combination of copeptin and cancer antigen 125 demonstrated higher diagnostic accuracy for acute HF in patients with AF. Both biomarkers were positively correlated with short-term cardiovascular events (15).

The aim of this prospective observational study was to compare serum levels of copeptin and cardiac biomarkers such as NT-proBNP and high-sensitivity cardiac troponin T (hsTnT) between patients with AF and a control group (CG), and to evaluate their associations with selected morphological and functional echocardiographic parameters. The second aim was to assess the role of copeptin in predicting AF recurrence and its association with all-cause mortality.

## 2 Materials and methods

### 2.1 Study design and population

A total of 100 patients with AF were included in this single-centre prospective observational study. The AF cohort comprised 44 men and 56 women, with a median age of 74.50 (67.75–82.00) years and an age range of 38–96 years. In all patients, AF was the primary reason for admission to the 1st Department of Internal Medicine, University Hospital in Košice. At the index admission, 55 patients had first-diagnosed AF, whereas 45 were admitted because of recurrent AF. Characteristics of AF and associated comorbidities were obtained from medical history, physical examination, and medical records.

The main inclusion criterion was clinical AF documented by a surface ECG recording in accordance with the 2020 European Society of Cardiology guidelines for the diagnosis and management of AF (18). Exclusion criteria included acute coronary syndrome (ACS), chronic kidney disease (CKD) stages G4 and G5 according to the Kidney Disease: Improving Global Outcomes (KDIGO) classification, end-stage renal disease (ESRD), liver failure, hyperthyroidism, diabetic ketoacidosis, and hyperosmolar hyperglycemic syndrome (19). The study was approved by the local Ethics Committee of the University Hospital in Košice, Slovakia, and all participants provided written informed consent.

The CG comprised 50 subjects, including 19 men and 31 women, with a median age of 62.50 (53.25–67.75) years and an age range of 43–89 years. Diagnostic, inclusion, and exclusion criteria for both groups are summarized in Table 1. Clinical characteristics, major comorbidities, clinical risk scores, rhythm restoration during the index hospitalization, and medication use at admission and discharge in the AF cohort are summarized in Supplementary Table 1. Persistent AF was the most frequent clinical pattern. Hypertension was the most common comorbidity, followed by congestive HF and previous stroke, transient ischemic attack, or systemic embolism. Beta-blockers and ACE inhibitors or angiotensin receptor blockers were the most frequently used medications at admission.

**Table 1:** Diagnostic, inclusion, and exclusion criteria for patients with AF and subjects in the CG.

Patients with AF
Inclusion criteria
• AF—supraventricular tachyarrhythmia characterized by uncoordinated atrial electrical activity resulting in ineffective atrial contraction. • Clinical AF—symptomatic or asymptomatic AF documented by surface ECG. The minimum duration of ECG monitoring required to confirm the diagnosis of clinical AF is at least 30 s or a complete 12-lead ECG recording. • AF as the cause of admission to the Department of Internal Medicine, University Hospital in Košice.
Exclusion criteria
• ACS • CKD stage G4 and G5 according to KDIGO, ESRD, and the need for extracorporeal renal replacement therapy • Liver failure • Hyperthyroidism • Diabetic ketoacidosis • Hyperosmolar hyperglycemic syndrome

Control group
Inclusion criteria
• Individuals aged over 40 years • Sinus rhythm
Exclusion criteria
• AF • Other supraventricular or ventricular arrhythmias • HFrEF • CKD stages G4–G5 according to KDIGO, ESRD, and the need for extracorporeal renal replacement therapy • Liver failure • Thyroid disorders • Adrenal gland disorders

ACS, acute coronary syndrome; AF, atrial fibrillation; CKD, chronic kidney disease; ECG, electrocardiogram; ESRD, end-stage renal disease; HFrEF, heart failure with reduced ejection fraction; KDIGO, kidney disease: improving global outcomes.

### 2.2 Laboratory investigations

Fasting blood samples were collected from all patients with AF and control subjects at 06:00 on the morning of the second study day after a fasting period of at least 8 h. Blood sampling was therefore standardized with respect to both timing and fasting status in both study groups. Standard biochemical measurements included serum sodium (S-Na), serum potassium (S-K), blood glucose, renal and hepatic parameters, thyroid-stimulating hormone (TSH), and C-reactive protein (CRP). All routine biochemical analyses were performed at the Department of Clinical Biochemistry, University Hospital in Košice.

Serum copeptin concentrations were measured using a commercially available coated-tube chemiluminescence sandwich immunoassay (B.R.A.H.M.S. AG, Hennigsdorf, Germany). Serum N-terminal pro-B-type natriuretic peptide (NT-proBNP) and high-sensitivity cardiac troponin T (hsTnT) concentrations were measured using electrochemiluminescence immunoassays on an Elecsys analyzer (Roche Diagnostics).

### 2.3 Echocardiography

Transthoracic echocardiography was performed by a single physician using an Esaote MyLab Twice ultrasound system equipped with a PA240 phased-array transducer. Image acquisition and cardiac chamber measurements followed established recommendations. The echocardiographic variables selected for statistical analysis were left atrial volume index (LAVi), right atrial area (RAA), tricuspid annular plane systolic excursion (TAPSE), left ventricular internal diameter at end-diastole indexed to body surface area (LVIDD/BSA), left ventricular mass index (LVMi), and left ventricular ejection fraction (LVEF) (20).

Left atrial volume (reference range, 27.68–58.82 mL) was measured at end-systole from the apical four- and two-chamber views using two-dimensional biplane assessment and was divided by BSA to obtain LAVi (reference range, 16–34 mL/m^2^). RAA (reference range, ≤18 cm^2^) was measured in the apical four-chamber view at end-systole immediately before tricuspid valve opening. TAPSE was determined by M-mode echocardiography from the apical four-chamber view, with the cursor aligned through the lateral tricuspid annulus; values <17 mm were considered indicative of reduced right ventricular systolic function (20).

Septal wall thickness at end-diastole (reference ranges, 0.6–1.0 cm in men and 0.6–0.9 cm in women), posterior wall thickness at end-diastole (reference ranges, 0.6–1.0 cm in men and 0.6–0.9 cm in women), and LVIDD (reference ranges, 4.2–5.8 cm in men and 3.8–5.2 cm in women) were measured in the parasternal long-axis view. Measurements were obtained perpendicular to the long axis of the left ventricle at the level of, or immediately below, the mitral valve leaflet tips. LVIDD was divided by BSA to obtain LVIDD/BSA, which was interpreted using sex- and BSA-specific reference values (20).

Left ventricular mass (reference ranges, 88–224 g in men and 67–162 g in women) was calculated using the Penn convention formula and divided by BSA to obtain LVMi (reference ranges, 49–115 g/m^2^ in men and 43–95 g/m^2^ in women). LVEF was measured from apical four- and two-chamber views using the biplane method of disks (modified Simpson's method); the reference ranges were 52%–72% in men and 54%–74% in women. The echocardiographic characteristics of the AF cohort are summarized in Table 2. Echocardiographic variables were not compared directly between the AF and control groups; their associations with copeptin and other biochemical and cardiac biomarkers were evaluated within the AF cohort (20).

**Table 2:** Selected morphological and functional echocardiographic characteristics of patients in the AF cohort (*n* = 100).

Variable	Median (Q1–Q3)
LAVi (mL/m^2^)	39.90 (29.91–53.00)
RAA (cm^2^)	19.76 (16.65–24.00)
TAPSE (mm)	19.80 (17.13–23.00)
LVIDD/BSA (cm/m^2^)	2.48 (2.28–2.68)
LVMi (g/m^2^)	109.42 (96.22–125.09)
LVEF (%)	54.50 (45.00–60.00)

LAVi, Left atrial volume index; LVEF, left ventricular ejection fraction; LVIDD/BSA, left ventricular internal diastolic diameter indexed to body surface area; LVMi, left ventricular mass index; RAA, right atrial area; TAPSE, tricuspid annular plane systolic excursion.

### 2.4 Atrial fibrillation follow-up

AF recurrence and all-cause mortality were evaluated during a 12-month follow-up period. AF recurrence was defined as the first documented reappearance of AF after successful restoration of sinus rhythm. Recurrence was assessed using hospital and outpatient medical records and was additionally verified by direct patient follow-up by telephone and at an in-person visit at 12 months. Both symptomatic and asymptomatic documented recurrences were included. AF recurrence required ECG documentation, either by a standard 12-lead ECG or, when symptom-triggered Holter monitoring was performed, by an AF episode lasting at least 30 s. Holter monitoring was not performed routinely and was used only in patients with symptoms suggestive of arrhythmia. Recurrences were counted from hospital discharge, without a blanking period.

AF was classified as paroxysmal in 24 patients, persistent in 48 patients, and permanent in 28 patients (Supplementary Graph 1). Sinus rhythm was restored during the index hospitalization in 62 patients: spontaneously in 28 patients (45.16%), by pharmacological cardioversion in 3 patients (4.84%), and by electrical cardioversion in 31 patients (50.00%).

These 62 patients were followed for AF recurrence over 12 months, during which recurrence occurred in 30 patients (48.39%). During the same follow-up period, 23 of the 100 patients with AF died (23.0%).

### 2.5 Statistical analysis

Statistical analyses were performed using JMP Student Edition, version 19 (SAS Institute Inc., Cary, NC, USA, RRID:SCR_014242). The distribution of continuous variables was assessed using the Shapiro–Wilk test. Because several variables were not normally distributed, continuous variables are presented as median (Q1–Q3), whereas categorical variables are presented as numbers and percentages. Between-group comparisons of continuous variables were performed using the two-sided Wilcoxon rank-sum test, which is equivalent to the Mann–Whitney U test. Categorical variables were compared using Pearson's chi-square test or Fisher's exact test, as appropriate. Associations between continuous variables were assessed using Spearman's rank correlation coefficient (*ρ*).

To account for differences in age and sex between the AF and CG, multivariable linear regression models were fitted using Standard Least Squares. Study group, age as a continuous variable, and sex as a categorical variable were included as independent variables. Right-skewed laboratory outcomes were log2-transformed and are reported as adjusted geometric mean ratios with 95% confidence intervals; eGFR was analysed on its original scale and is reported as an adjusted mean difference. A study group-by-sex interaction was additionally tested for copeptin. Model assumptions were assessed using residual plots and examination of influential observations.

A multivariable binary logistic regression model was used to identify predictors of AF recurrence. Copeptin was retained as the principal biomarker of interest, while age, LAVi, and LVEF were selected on clinical grounds for the revised analysis as measures representing demographic risk, atrial structural remodelling, and left ventricular systolic function, respectively. All four variables were entered simultaneously, and their effects were evaluated using likelihood-ratio chi-square tests. For copeptin, the principal biomarker of interest, the adjusted odds ratio with a 95% confidence interval was reported per 10-pmol/L increase. ROC curve analysis was additionally performed for copeptin alone among patients included in the multivariable analysis. The optimal copeptin cut-off was selected by maximizing the Youden index.

## 3 Results

### 3.1 Comparison between patients with atrial fibrillation and the control group

Clinical and biochemical characteristics of patients with AF and the CG are summarized in Table 3.

**Table 3:** Comparison of measured parameters in patients with AF and in the CG.

Variable	AF (*n* = 100) Median (Q1–Q3)	CG (*n* = 50) Median (Q1–Q3)	*p*-value
Male sex (n, %)	44 (44.0)	19 (38.0)	0.4828
Age (years)	74.50 (67.75–82.00)	62.50 (53.25–67.75)	**<0.0001**
Serum glucose (mmol/L)	7.10 (6.18–9.10)	5.10 (4.40–5.69)	**<0**.**0001**
S-Na (mmol/L)	135.75 (133.08–137.40)	137.60 (135.85–139.28)	**<0**.**0001**
S-K (mmol/L)	4.00 (3.70–4.30)	4.35 (4.20–4.65)	**<0**.**0001**
S-Osm (mOsm/kg)	286.40 (281.71–292.43)	289.22 (282.74–292.81)	0.2509
eGFR (mL/min/1.73 m^2^)	70.00 (53.75–86.25)	83.50 (66.25–100.00)	**0**.**0003**
CRP (mg/L)	10.99 (3.15–42.52)	2.23 (1.12–4.18)	**<0**.**0001**
hsTnT (*μ*g/L)	0.020 (0.012–0.036)	0.007 (0.005–0.010)	**<0**.**0001**
NT-proBNP (ng/L)	2,279.50 (992.85–6,716.25)	62.41 (30.11–182.70)	**<0**.**0001**
Copeptin (pmol/L)	14.52 (6.98–36.83)	4.10 (2.70–6.88)	**<0**.**0001**

AF, atrial fibrillation; CG, control group; CRP, C-reactive protein; eGFR, estimated glomerular filtration rate; hsTnT, high-sensitivity cardiac troponin T; NT-proBNP, N-terminal pro-B-type natriuretic peptide; S-K, serum potassium; S-Na, serum sodium; S-Osm, serum osmolality.

Bold values indicate statistically significant results (*p* < 0.05).

Sex distribution did not differ significantly between the groups (*p* = 0.4828). Compared with the CG, patients with AF were older and had higher serum glucose, CRP, hsTnT, and NT-proBNP concentrations, whereas S-Na, S-K, and eGFR were lower (all *p* ≤ 0.0003). Serum osmolality (S-Osm) did not differ significantly between the groups (*p* = 0.2509). Serum copeptin concentrations were higher in the AF group than in the CG, with median values of 14.52 (6.98–36.83) and 4.10 (2.70–6.88) pmol/L, respectively (*p* < 0.0001).

After adjustment for age and sex, serum copeptin concentrations remained significantly higher in patients with AF than in the CG (adjusted geometric mean ratio, 2.995; 95% CI, 2.040–4.398; *p* < 0.0001). Significant between-group differences also remained for NT-proBNP (adjusted geometric mean ratio, 17.960; 95% CI, 10.605–30.418; *p* < 0.0001), hsTnT (adjusted geometric mean ratio, 2.375; 95% CI, 1.745–3.233; *p* < 0.0001), and CRP (adjusted geometric mean ratio, 4.110; 95% CI, 2.336–7.232; *p* < 0.0001). In contrast, the between-group difference in eGFR was attenuated after adjustment for age and sex and was no longer statistically significant (adjusted mean difference, AF minus CG, −6.96 mL/min/1.73 m^2^; 95% CI, −15.06–1.14; *p* = 0.0915). Male sex was independently associated with higher serum copeptin concentrations (adjusted geometric mean ratio, 1.675; 95% CI, 1.193–2.353; *p* = 0.0031). However, the study group-by-sex interaction was not statistically significant (*p* = 0.347), indicating that the difference in copeptin concentrations between patients with AF and the CG did not vary significantly by sex.

### 3.2 Correlations between copeptin and biochemical parameters

Correlations between serum copeptin and biochemical parameters in patients with AF and the CG are presented in Table 4.

**Table 4:** Correlations of copeptin with biochemical parameters in patients with AF and in the CG.

Variable		Copeptin—AF (*n* = 100)	Copeptin—CG (*n* = 50)
Serum glucose (mmol/L)	*ρ*	−0.1100	**0**.**3725**
	p	0.2809	**0**.**0077**
S-Na (mmol/L)	ρ	−0.0417	**0**.**4622**
	p	0.6837	**0**.**0007**
S-K (mmol/L)	ρ	−0.0145	−0.2025
	p	0.8875	0.1584
S-Osm (mOsm/kg)	ρ	0.1771	**0**.**5142**
	p	0.0811	**0**.**0001**
eGFR (mL/min/1.73 m^2^)	ρ	**−0**.**3231**	−0.0597
	p	**0**.**0012**	0.6803
CRP (mg/L)	ρ	**0**.**4862**	0.1871
	p	**<0**.**0001**	0.1932
hsTnT (μg/L)	ρ	**0**.**4904**	0.2579
	p	**<0**.**0001**	0.0706
NT-proBNP (ng/L)	ρ	**0**.**3458**	0.2008
	p	**0**.**0005**	0.1620

AF, atrial fibrillation; CG, control group; CRP, C-reactive protein; eGFR, estimated glomerular filtration rate; hsTnT, high-sensitivity cardiac troponin T; NT-proBNP, N-terminal pro-B-type natriuretic peptide; S-K, serum potassium; S-Na, serum sodium; S-Osm, serum osmolality.

Bold values indicate statistically significant results (*p* < 0.05).

In the AF group, copeptin showed positive correlations with CRP (ρ = 0.4862, *p* < 0.0001), hsTnT (ρ = 0.4904, *p* < 0.0001), and NT-proBNP (ρ = 0.3458, *p* = 0.0005), and an inverse correlation with eGFR (ρ = −0.3231, *p* = 0.0012). No significant correlations were observed between copeptin and serum glucose, S-Na, S-K, or S-Osm.

In the CG, significant positive correlations were observed between copeptin and serum glucose (ρ = 0.3725, *p* = 0.0077), S-Na (ρ = 0.4622, *p* = 0.0007), and S-Osm (*ρ* = 0.5142, *p* = 0.0001). The correlation between copeptin and hsTnT did not reach statistical significance (ρ = 0.2579, *p* = 0.0706).

### 3.3 Correlations between biochemical markers and echocardiographic parameters

Correlations between selected biochemical markers and echocardiographic parameters in patients with AF are presented in Table 5.

**Table 5:** Correlations between humoral and selected echocardiographic parameters in patients with AF.

Variable		LAVi	RAA	TAPSE	LVIDD/BSA	LVMi	LVEF
S-Na	ρ	−0.1913	−0.1470	0.1264	**−0**.**3029**	**−0**.**2808**	0.0833
	p	0.0813	0.1992	0.2405	**0**.**0032**	**0**.**0067**	0.4248
S-K	ρ	−0.0858	−0.0389	−0.0313	0.0876	0.0174	**−0**.**2668**
	p	0.4376	0.7355	0.7720	0.4035	0.8689	**0**.**0093**
S-Osm	ρ	−0.0293	−0.0779	−0.0934	−0.1934	−0.0308	−0.0693
	p	0.7911	0.4981	0.3866	0.0633	0.7706	0.5067
eGFR	ρ	−0.1136	−0.1225	**0**.**3499**	0.0724	0.0144	0.1698
	p	0.3036	0.2854	**0**.**0008**	0.4902	0.8919	0.1019
CRP	ρ	**0**.**3528**	0.2129	−0.1924	0.1914	**0**.**3707**	**−0**.**2204**
	p	**0**.**0010**	0.0613	0.0725	0.0660	**0**.**0003**	**0**.**0328**
hsTnT	ρ	**0**.**3710**	0.1906	**−0**.**2434**	0.1345	**0**.**2516**	**−0**.**2114**
	p	**0**.**0005**	0.0947	**0**.**0223**	0.1987	**0**.**0155**	**0**.**0408**
NT-proBNP	ρ	**0**.**5449**	**0**.**3591**	**−0**.**3216**	**0**.**3545**	**0**.**3722**	**−0**.**3793**
	p	**<0**.**0001**	**0**.**0012**	**0**.**0022**	**0**.**0005**	**0**.**0003**	**0**.**0002**
Copeptin	ρ	**0**.**2598**	0.0757	**−0**.**2277**	0.0071	**0**.**2252**	−0.1404
	p	**0**.**0177**	0.5102	**0**.**0339**	0.9465	**0**.**0319**	0.1795

CRP, C-reactive protein; eGFR, estimated glomerular filtration rate; hsTnT, high-sensitivity cardiac troponin T; LAVi, left atrial volume index; LVEF, left ventricular ejection fraction; LVIDD/BSA, left ventricular internal diastolic diameter indexed to body surface area; LVMi, left ventricular mass index; NT-proBNP, N-terminal pro-B-type natriuretic peptide; RAA, right atrial area; S-K, serum potassium; S-Na, serum sodium; S-Osm, serum osmolality; TAPSE, tricuspid annular plane systolic excursion.

Bold values indicate statistically significant results (*p* < 0.05).

Spearman's rank correlation analysis demonstrated inverse correlations between S-Na and LVIDD/BSA (ρ = −0.3029, *p* = 0.0032) and between S-Na and LVMi (ρ = −0.2808, *p* = 0.0067). S-K was inversely correlated with LVEF (ρ = −0.2668, *p* = 0.0093), whereas eGFR was positively correlated with TAPSE (ρ = 0.3499, *p* = 0.0008). No significant correlations were observed between S-Osm and the assessed echocardiographic parameters.

CRP showed positive correlations with LAVi (ρ = 0.3528, *p* = 0.0010) and LVMi (ρ = 0.3707, *p* = 0.0003) and an inverse correlation with LVEF (ρ = −0.2204, *p* = 0.0328). HsTnT was positively correlated with LAVi (ρ = 0.3710, *p* = 0.0005) and LVMi (ρ = 0.2516, *p* = 0.0155) and inversely correlated with TAPSE (ρ = −0.2434, *p* = 0.0223) and LVEF (ρ = −0.2114, *p* = 0.0408).

NT-proBNP was significantly correlated with all assessed morphological and functional echocardiographic parameters, including positive correlations with LAVi (ρ = 0.5449, *p* < 0.0001), RAA (ρ = 0.3591, *p* = 0.0012), LVIDD/BSA (ρ = 0.3545, *p* = 0.0005), and LVMi (ρ = 0.3722, *p* = 0.0003), and inverse correlations with TAPSE (ρ = −0.3216, *p* = 0.0022) and LVEF (ρ = −0.3793, *p* = 0.0002). Serum copeptin concentrations were positively correlated with LAVi (ρ = 0.2598, *p* = 0.0177) and LVMi (ρ = 0.2252, *p* = 0.0319) and inversely correlated with TAPSE (ρ = −0.2277, *p* = 0.0339).

### 3.4 Atrial fibrillation recurrence and mortality

Patients were followed for AF recurrence and all-cause mortality over a 12-month period following hospital discharge.

Among the 62 patients in whom sinus rhythm was restored during the index hospitalization, AF recurrence was assessed using a multivariable binary logistic regression model including copeptin, age, LAVi, and LVEF. Each 10-pmol/L increase in copeptin was independently associated with higher odds of AF recurrence after adjustment for age, LAVi, and LVEF (adjusted OR, 1.61; 95% CI, 1.01–2.56; Wald *p* = 0.044). The overall likelihood-ratio effect of copeptin was also statistically significant (likelihood-ratio *χ*^2^ = 7.915; *p* = 0.0049; Supplementary Graph 2). Likelihood-ratio testing also showed statistically significant effects of LVEF (*p* < 0.0001) and LAVi (*p* = 0.0028), whereas the effect of age was not statistically significant (*p* = 0.0868; Supplementary Graph 2). Among patients included in the multivariable analysis, ROC analysis of copeptin alone yielded an AUC of 0.674 (95% CI, 0.526–0.821). A cut-off of 11.68 pmol/L provided a sensitivity of 63.33% and a specificity of 75.00% (Supplementary Graph 3).

Non-survivors had higher serum copeptin concentrations than survivors, with median values of 35.35 (10.35–56.82) and 12.20 (6.54–29.43) pmol/L, respectively (*p* = 0.0051). Non-survivors also had higher hsTnT concentrations, 0.040 (0.023–0.090) versus 0.017 (0.011–0.030) μg/L (*p* < 0.0001), and higher NT-proBNP concentrations, 4,379 (1,852–15,261) versus 2,015 (910.9–6,276) ng/L (*p* = 0.0198). In contrast, eGFR was lower in non-survivors than in survivors, with median values of 50.0 (27.5–72.5) and 73.0 (57.0–87.0) mL/min/1.73 m^2^, respectively (*p* = 0.0081).

## 4 Discussion

Compared with the CG, patients with AF had significantly lower serum sodium and higher serum NT-proBNP and copeptin concentrations, suggesting a potential interplay between hyponatremia, HF, and AF. Activation of the AVP system, reflected by circulating copeptin, may explain the association between hyponatremia and AF in HF (21–23). Importantly, serum copeptin concentrations remained significantly higher in patients with AF than in the CG after adjustment for age and sex, indicating that the between-group difference was not fully explained by the older age of the AF cohort. Previous studies have reported higher circulating copeptin concentrations in men than in women. Consistent with this evidence, male sex was independently associated with higher copeptin concentrations in our adjusted analysis. However, the group-by-sex interaction was not significant, indicating that the difference between patients with AF and the CG did not vary significantly by sex (5, 24, 25).

In a multicenter observational study of patients with HFrEF, Cavusoglu et al. reported an AF prevalence of 33.3% in those with hyponatremia, significantly higher than the 18.8% observed in normonatremic patients. In multivariable logistic regression, hyponatremia remained significantly and independently associated with AF occurrence (OR, 2.457; 95% CI, 1.586–3.806; *p* < 0.001), alongside other well-established risk factors for AF. The proportion of patients with advanced NYHA class III–IV was higher among those with hyponatremia. Notably, AF prevalence remained significantly higher in hyponatremic than in normonatremic patients within NYHA classes I and II (21). Moreover, in a prospective cohort study by Zhou et al., both hyponatremia (HR, 2.19; 95% CI, 1.50–3.20; *p* < 0.001) and hypernatremia (HR, 4.03; 95% CI, 2.32–7.02; *p* < 0.001) at hospital admission were associated with an increased risk of all-cause mortality within 365 days after discharge in patients with AF without HF. Both associations remained significant after multivariable adjustment for age, diabetes mellitus, and medication use (26).

Several AVP-mediated mechanisms may link hyponatremia and AF in patients with HF. Through V2 receptor activation, AVP induces hypervolemia and thereby increases atrial wall stretch. Hypervolemic hyponatremia may also alter the electrical properties of the sinoatrial node and pulmonary veins. V1a receptor activation may modify intracellular calcium homeostasis in cardiomyocytes and trigger ectopic activity in the pulmonary veins. Increased AF incidence has also been reported in patients with HF receiving vasopressor therapy, including vasopressin, for vasoplegic shock after cardiac surgery and in patients with septic shock receiving prolonged vasopressin therapy. Combined V1a/V2 receptor blockade represents a potential therapeutic approach, but evidence of its clinical benefit remains limited (23, 27, 28).

In the CG, copeptin was significantly positively correlated with S-Osm, whereas no significant association with S-Osm or S-Na was observed in patients with AF. We hypothesize that this finding may be attributed to concomitant HF and non-osmotic stimulation of AVP secretion. This interpretation is consistent with previous findings in hospitalized patients and HF populations (29–31). Patients with AF and altered fluid balance, particularly those with concomitant HF, may be exposed to multiple non-osmotic stimuli of AVP release compared with individuals with stable fluid balance. Altered renal water handling and diuretic use may also have attenuated the association between copeptin and osmotic markers. However, because urine osmolality was not measured, this mechanistic interpretation remains speculative (32).

Consistent with our findings, Avci et al. demonstrated positive correlations between copeptin and both hs-CRP and NT-proBNP in a prospective study of patients with mild-to-moderate rheumatic mitral stenosis. Copeptin was also inversely correlated with mitral valve area and positively correlated with LAVi. In multivariable logistic regression, copeptin independently predicted paroxysmal AF (OR, 2.81; 95% CI, 1.3–5.29; *p* < 0.001) (3).

Elevated hsTnT concentrations in patients with AF compared with the CG are consistent with population-based evidence linking troponin elevation to incident AF. Associations were reported across several cohorts, including a 4.8-fold adjusted risk in the study by Anegawa et al., a 1.78-fold risk at hsTnT concentrations ≥14 ng/L in ARIC, and a relationship between baseline hsTnT and incident AF during 11.2 years of median follow-up in the Cardiovascular Health Study. In our study, copeptin was positively correlated with hsTnT in patients with AF, whereas the corresponding association in the CG was not statistically significant. AF may be accompanied by low-grade cardiomyocyte injury and troponin release from tachyarrhythmia-associated myocardial and hemodynamic stress, even without significant coronary artery disease. The same hemodynamic and systemic stressors, including hypoxia and concomitant HF, may stimulate non-osmotic AVP secretion and increase circulating copeptin concentrations. The observed correlation may therefore reflect parallel responses of copeptin and hsTnT to a common pathophysiological milieu rather than a direct causal relationship. Although the role of copeptin as a myocardial injury biomarker alongside hsTnT in acute myocardial infarction remains controversial, our findings suggest that copeptin may provide complementary information on myocardial and neurohumoral stress and may reflect a higher-risk AF phenotype, including greater susceptibility to recurrence and adverse clinical outcomes (5, 22, 33).

In our cohort, copeptin concentrations were weakly positively correlated with LAVi and LVMi. These findings suggest that higher copeptin concentrations may accompany atrial enlargement and increased left ventricular mass in patients with AF, consistent with structural remodeling and hemodynamic stress. The association with LAVi is consistent with the findings of Avci et al. in patients with rheumatic mitral stenosis. A reduction in copeptin concentrations during the first 24 h after percutaneous balloon mitral valvuloplasty has also been described, supporting a link between the AVP–copeptin system, intra-atrial pressure, and atrial wall stretch. Together, these observations suggest that elevated copeptin may reflect structural and hemodynamic atrial remodeling and be associated with greater susceptibility to AF recurrence (3). Copeptin concentrations also showed a weak inverse correlation with right ventricular (RV) longitudinal systolic function, assessed by TAPSE. Consistent with our findings, Harbrücker et al. reported progressively higher copeptin concentrations with decreasing TAPSE in a prospective single-centre controlled study. Their cohort excluded patients with LVEF <50%, moderate-to-severe valvular heart disease, and relevant pulmonary arterial hypertension, although 43% of participants had AF. Copeptin showed good discrimination for reduced RV longitudinal function at TAPSE thresholds of <18 mm (AUC, 0.793) and <14 mm (AUC, 0.801). In a multivariable linear regression model adjusted for age, sex, creatinine, and NT-proBNP, copeptin remained independently associated with TAPSE, whereas NT-proBNP did not, suggesting that copeptin may reflect impaired RV longitudinal function (34).

In multivariable logistic regression, copeptin was identified as an independent predictor of 12-month AF recurrence after restoration of sinus rhythm. Each 10-pmol/L increase in copeptin was associated with 61% higher odds of recurrence after adjustment for age, LAVi, and LVEF (adjusted OR, 1.61; 95% CI, 1.01–2.56). However, ROC analysis of copeptin alone showed modest discriminatory ability (AUC, 0.674; 95% CI, 0.526–0.821). In a different clinical setting, Avci et al. reported that higher copeptin concentrations independently predicted paroxysmal AF in patients with rheumatic mitral stenosis (3). In the GISSI-AF biomarker substudy, copeptin was assessed but did not independently predict AF recurrence, whereas hsTnT and NT-proBNP did (35). Similarly, Schnabel et al. found no independent association between copeptin and prevalent AF in a population-based cross-sectional study (36). More recently, the EAST-AFNET 4 biomolecule study showed that lower baseline concentrations of NT-proBNP, BMP10, and ANGPT2 predicted sinus rhythm during follow-up, highlighting the emerging role of multipathway biomarker profiling in rhythm prediction (11). The SMURF study showed that copeptin increased approximately sixfold immediately after radiofrequency ablation and returned to near-baseline concentrations by the following day, a pattern more consistent with an acute systemic neurohumoral response to the procedure than with a marker of acute procedural efficacy or reduced arrhythmic burden (37).

A significant finding of our study was the association between higher serum copeptin concentrations and 12-month all-cause mortality in patients hospitalized for AF. Although multiple studies have linked copeptin to adverse outcomes, including mortality, across various clinical conditions, our findings extend this evidence to hospitalized patients with AF, with all-cause mortality evaluated as a separate endpoint (14). Copeptin reflects a nonspecific neuroendocrine stress response, while low concentrations have been evaluated in early rule-out strategies for acute myocardial infarction and have been associated with a lower likelihood of adverse outcomes in respiratory infections (5). Recent evidence also supports the prognostic relevance of copeptin in decompensated HF, while studies in community-based, diabetic, and septic populations have linked elevated concentrations to mortality or greater disease severity (6, 24, 38–40). In our cohort, non-survivors also had higher hsTnT and NT-proBNP concentrations and lower eGFR, suggesting that elevated copeptin may reflect greater cardiorenal, hemodynamic, and systemic disease burden. Although renal dysfunction may influence circulating copeptin, some studies in cardiorenal populations suggest that its prognostic relevance may not be entirely explained by impaired kidney function (5, 24, 39).

This study has several limitations. First, its prospective observational design and relatively small sample size may limit generalizability. Although the multivariable recurrence model was restricted to four clinically selected predictors, recurrence events remained limited; therefore, overfitting cannot be excluded, and the findings should be confirmed in larger independent cohorts. Second, the CG was not age-matched, introducing potential confounding. To address this, between-group differences were evaluated using multivariable models adjusted for age and sex. Differences in copeptin, NT-proBNP, hsTnT, and CRP remained statistically significant after adjustment, whereas the eGFR difference was attenuated and no longer significant. Eligibility criteria also differed between the groups: HFrEF was excluded from the CG, whereas HF was not an exclusion criterion in the AF cohort, potentially introducing selection bias and residual confounding. Although sex was incorporated into the multivariable analyses and no significant study group-by-sex interaction was observed, the sample size limited detailed sex-stratified analyses. Third, copeptin was measured only once during the index hospitalization, without assessment of dynamic changes during follow-up or after restoration of sinus rhythm. Despite these limitations, the strengths of our study include its prospective design, simultaneous analysis of copeptin with established cardiac biomarkers and echocardiographic parameters, and evaluation of both recurrence and mortality in a clinically well-defined cohort. To our knowledge, this is the first study to demonstrate that, in patients hospitalized for AF, elevated serum copeptin independently predicts recurrence and is associated with increased all-cause mortality over a 12-month period.

## 5 Conclusions

In this prospective observational study, serum copeptin concentrations were higher in hospitalized patients with AF than in the CG after adjustment for age and sex and were associated with NT-proBNP, hsTnT, CRP, and echocardiographic parameters, including LAVi, LVMi, and TAPSE. Higher copeptin independently predicted 12-month AF recurrence after restoration of sinus rhythm and was associated with increased all-cause mortality. These findings suggest that copeptin reflects neurohumoral stress, myocardial injury, inflammation, and structural and hemodynamic cardiac burden, and may serve as a useful biomarker for risk stratification and prognostic assessment in hospitalized patients with AF.

## Data Availability

The original contributions presented in the study are included in the article/Supplementary Material, further inquiries can be directed to the corresponding author.
