# Supplementary material for: Serum copeptin predicts atrial fibrillation recurrence and is associated with increased all-cause mortality
Source: Front Cardiovasc Med. 2026 Aug 25;13:1859765. doi: 10.3389/fcvm.2026.1859765 (PMC13550959; doi:10.3389/fcvm.2026.1859765)
Supplement: Supplementary file 1 [file Datasheet1.docx]

Supplementary Material

**Supplementary Table 1** Clinical characteristics, comorbidities, rhythm management, and medication use in patients with AF (n = 100).

| **Variable** | **Value** |
| --- | --- |
| **AF presentation at index admission** | |
| First-diagnosed AF | 55 (55.0) |
| Recurrent AF at index admission | 45 (45.0) |
| **AF clinical pattern** | |
| Paroxysmal AF | 24 (24.0) |
| Persistent AF | 48 (48.0) |
| Permanent AF | 28 (28.0) |
| **Clinical symptom score** | |
| EHRA class 1 | 23 (23.0) |
| EHRA class 2a | 8 (8.0) |
| EHRA class 2b | 18 (18.0) |
| EHRA class 3 | 27 (27.0) |
| EHRA class 4 | 24 (24.0) |
| **Clinical risk scores** | |
| CHA₂DS₂-VASc score | 5 (4–6) |
| HAS-BLED score | 2 (2–3) |
| **Comorbidities** | |
| Congestive heart failure | 51 (51.0) |
| Hypertension | 89 (89.0) |
| Diabetes mellitus | 28 (28.0) |
| Abnormal renal function (HAS-BLED criterion) | 12 (12.0) |
| Previous stroke, TIA, or systemic embolism | 34 (34.0) |
| Previous myocardial infarction | 17 (17.0) |
| Peripheral artery disease | 21 (21.0) |
| **Rhythm restoration during the index hospitalization** | |
| Restoration of sinus rhythm | 62 (62.0) |
| Spontaneous conversion | 28/62 (45.2) |
| Pharmacological cardioversion with amiodarone | 3/62 (4.8) |
| Electrical cardioversion | 31/62 (50.0) |
| **Medication use at admission** | |
| ACE inhibitor or ARB | 60 (60.0) |
| ARNI | 2 (2.0) |
| Beta-blocker | 63 (63.0) |
| Rate-limiting calcium channel blocker | 3 (3.0) |
| Antiarrhythmic drug | 22 (22.0) |
| Amiodarone | 15 (15.0) |
| Propafenone | 7 (7.0) |
| Digoxin | 6 (6.0) |
| Diuretic | 42 (42.0) |
| Mineralocorticoid receptor antagonist | 10 (10.0) |
| Lipid-lowering therapy | 46 (46.0) |
| **Medication use at discharge** | |
| ACE inhibitor or ARB | 58 (58.0) |
| ARNI | 2 (2.0) |
| Beta-blocker | 91 (91.0) |
| Rate-limiting calcium channel blocker | 3 (3.0) |
| Antiarrhythmic drug | 33 (33.0) |
| Amiodarone | 20 (20.0) |
| Propafenone | 13 (13.0) |
| Digoxin | 12 (12.0) |
| Diuretic | 48 (48.0) |
| Mineralocorticoid receptor antagonist | 17 (17.0) |
| Lipid-lowering therapy | 52 (52.0) |

Data are presented as n (%), n/N (%), or median (Q1–Q3), as appropriate. ACE, angiotensin-converting enzyme; AF, atrial fibrillation; ARB, angiotensin receptor blocker; ARNI, angiotensin receptor–neprilysin inhibitor; EHRA, European Heart Rhythm Association; TIA, transient ischemic attack.

Supplementary Graph 1 Types of atrial fibrillation according to sex.

Alt text: Bar chart comparing the distribution of atrial fibrillation types between male and female patients, showing the numbers of patients with paroxysmal, persistent, and permanent atrial fibrillation in each sex group.

Supplementary Graph 2 Effect summary of the multivariable binary logistic regression model for 12-month AF recurrence including copeptin, age, LAVi, and LVEF. AF, atrial fibrillation; LAVi, left atrial volume index; LVEF, left ventricular ejection fraction.

Alt text: Effect summary displaying LogWorth values and likelihood-ratio p-values for copeptin, age, LAVi, and LVEF entered simultaneously into the multivariable binary logistic regression model for 12-month AF recurrence.

Supplementary Graph 3 ROC curve for copeptin levels in patients with AF included in the multivariable analysis—cut-off, 11.68 pmol/L; sensitivity, 63.33%; specificity, 75.00%; AUC, 0.674; 95% CI, 0.526–0.821. AF, atrial fibrillation; AUC, area under the curve; CI, confidence interval; ROC, receiver operating characteristic.

**Alt text:** ROC curve showing the ability of copeptin levels to discriminate 12-month AF recurrence among patients included in the multivariable analysis.
